# ADAMTS9-AS1 Constrains Breast Cancer Cell Invasion and Proliferation via Sequestering miR-301b-3p

**DOI:** 10.3389/fcell.2021.719993

**Published:** 2021-11-24

**Authors:** Junqing Chen, Ling Cheng, Weibin Zou, Rong Wang, Xiaojia Wang, Zhanhong Chen

**Affiliations:** ^1^Department of Breast Medical Oncology, The Cancer Hospital of the University of Chinese Academy of Sciences, Zhejiang Cancer Hospital, Hangzhou, China; ^2^Institute of Basic Medicine and Cancer (IBMC), Chinese Academy of Sciences, Hangzhou, China; ^3^Shanghai Engineering Research Center of Pharmaceutical Translation, Shanghai, China

**Keywords:** ADAMTS9-AS1, miR-301b-3p, TGFBR2, JAK STAT signaling pathway, breast cancer

## Abstract

**Objective:** For determination of how ADAMTS9-AS1/miR-301b-3p/TGFBR2/JAK STAT signaling axis modulates progression of breast cancer cells.

**Methods:** Target lncRNA was determined by differential analysis of breast cancer expression data and survival analysis. Differentially expressed miRNAs and target mRNAs that had binding sites with target lncRNA were predicted. GSEA software was used to carry out pathway enrichment analysis for mRNAs. Binding of the researched genes were tested with RNA binding protein immunoprecipitation (RIP). How miR-301b-3p bound TGFBR2 mRNA was tested by dual-luciferase method. Transwell, colony formation, EdU approaches were employed for verification of invasion and proliferation of breast cancer cells in each treatment group.

**Results:** Markedly inactivated ADAMTS9-AS1 in breast cancer pertained to patient’s prognosis. MiR-301b-3p was capable of binding TGFBR2/ADAMTS9-AS1. However, overexpression of ADAMTS9-AS1 stimulated miR-301b-3p binding ADAMTS9-AS1 and repressed miR-301b-3p binding TGFBR2 mRNA. ADAMTS9-AS1 interference enhanced cancer proliferation and invasion, facilitated levels of KI67, PCNA, MMP-9 and MMP-2, and activated the JAK STAT signaling pathway. While silencing miR-301b-3p reversed the effect of ADAMTS9-AS1 interference. In addition, TGFBR2 interference or restraining JAK STAT signaling counteracted the effect of ADAMTS9-AS1.

**Conclusion:** ADAMTS9-AS1 could sequester miR-301b-3p to inhibit progression of breast cancer via TGFBR2/JAK STAT pathway. This study supplies a rationale for incremental apprehension of ADAMTS9-AS1 in breast cancer progression.

## Introduction

As the most prevalent female malignant tumor globally, morbidity of breast cancer has been steadily increasing since 1987, and its incidence accounts for approximately 24.5% of all women’s malignancies (Sung et al., 2021). Not a single-gene disorder, breast cancer is a diversified disease classified by histology, immunopathology, mRNA expression profile and miRNA expression features rather than a monogenic disease (Laulin and Brudieux, 1990). As molecular biological research deepens, abnormal signaling pathways of tumor inhibitor genes and oncogenes manifest connection with breast cancer development (Boo et al., 2016; Gilam et al., 2016; Nassar et al., 2017; Sun et al., 2017). So far, the specific molecular mechanism for breast cancer progression has not been completely clarified.

Long non-coding RNAs (lncRNAs) exert a role in expression modulation at genetic levels and also exert functions on tumor-relevant cancer cell behaviors (Mercer et al., 2009; Fatica and Bozzoni, 2014). More and more evidence demonstrated association lncRNA-miRNA-mRNA axis maintains in tumor development. As a critical post-transcriptional regulator, the activity of miRNA can be sequestered by lncRNA (Wang et al., 2018; Peng et al., 2019; Shen et al., 2019). Such lncRNA is called competitive endogenous RNA (ceRNA). ADAMTS9-AS1 is an antisense lncRNA generated by reverse transcription of protein-coding gene ADAMTS9 and closely linked with various tumors. ADAMTS9 is a tumor inhibitor in assorted cancer cells: esophagus carcinoma (Lo et al., 2007), gastric cancer (Du et al., 2013), breast cancer (Ocak et al., 2013) and nasopharyngeal carcinoma (Lung et al., 2008; Sheu et al., 2009). Nevertheless, there is a shortage of deep research about the function of lncRNA ADAMTS9-AS1 in tumors. ADAMTS9-AS1 was found in esophageal squamous cell carcinoma at first (Li et al., 2017). It is greatly important for ectoderm and epithelial cells, as well as a novel biomarker for esophageal squamous cell carcinoma (Li et al., 2017). A study comprehensively analyzed lncRNA and found that ADAMTS9-AS1 is central in differential co-expression network in ovarian cancer (Wang et al., 2016). Few references have deeply researched ADAMTS9-AS1 in breast cancer yet. Here, such work was performed, which might conduce to biological understanding of this lethal disease.

Here, on the basis of the downloaded bioinformatics data, LncRNA ADAMTS9-AS1 was determined using differential analysis as well as survival analysis. Its role in breast cancer was explored by cell functional experiments. In an effort to scrutinize its downstream mechanism, bioinformatics databases were applied to predict differential miRNA and its target gene that had binding sites with ADAMTS9-AS1. The evidence proved that ADAMTS9-AS1 restrained invasion and proliferation of cancer cells through sequestering miR-301b-3p to target TGFBR2 and regulate JAK STAT signaling pathway. The results provide a foundation for further revealing of the mechanism of ADAMTS9-AS1 in breast cancer.

## Materials and Methods

### Bioinformatics Analysis

The Cancer Genome Atlas (TCGA)^1^ provided expression data of lncRNAs, mRNAs and miRNAs related to breast cancer. Clinical information was offered in Supplementary Table 1. Differential expression analysis was performed with edgeR (| logFC| > 2, *padj* < 0.05). Differentially expressed lncRNAs (DElncRNAs) and miRNAs (DEmiRNAs) that had binding sites were screened through miRcode.^2^ Downstream regulatory target genes of DEmiRNAs were predicted by miRDB,^3^ TargetScan,^4^ along with miRTarBase.^5^ The predicted mRNAs were intersected with DEmRNAs to obtain target DEmRNAs of DEmiRNAs. Pathway enrichment analysis of unigenes was conducted by using Gene Set Enrichment Analysis (GSEA).

### Cell Culture and Transfection

Mammary epithelial cells MCF10A (CBP60419) and breast cancer cells MDA-MB-231 (CBP60382), BT-549 (CBP60357), MDA-MB-468 (CBP60387) and MCF-7 (CBP60380) were cultured in complete mediums. All mediums and cell lines were purchased from Nanjing Cobioer Biotech. Cells were inoculated in 6-well plates at 3 × 10^5^ each well until cell growth density reached 70–90%. Then, cells were subjected to transfection utilizing Lipofectamine 2000 (11668-019, Invitrogen, California, United States). Next, 250 μl Opti-MEM (11668-019, Invitrogen, California, United States) medium was used to release 4 μg target plasmids and 10 μl Lipofectamine 2000, respectively, and mixed well by flicking. After being maintained at room temperature for 5 min, two liquids were mixed evenly, and added into cell culture wells after 20 min. Culture plates were shaken gently and mixed evenly. Then plates were generally cultured in an incubator. After 24 h, medium was changed and cells were collected 36–48 h later.

The si-ADAMTS9-AS1 and si-TGFBR2 were chemically synthesized by Genepharma (Shanghai, China). The siRNA transfection was undertaken with Lipofectamine 2000 (Invitrogen) as the standard plan. The oe-negative control (NC) (NC plasmids of overexpressing ADAMTS9-AS1, the vector used was pcDNA*™*3.1(+) vector (V79020), Invitrogen), oe-ADAMTS9-AS1 (overexpressing ADAMTS9-AS1 plasmids, the vector used was pcDNA*™*3.1(+) vector), inhibitor NC (NC of miR-301b-3p inhibitor) and miR-301b-3p inhibitor were procured from Genepharma (Shanghai, China).

### Quantitative Reverse Transcription-Polymerase Chain Reaction

Total RNA was detached from cells (1 × 10^6^ per well) with Trizol (No. 16096020, Thermo Fisher Scientific, New York, United States). Next, 5 μg samples were chosen for reverse transcription to synthesis into cDNA following the instruction of cDNA kit (K1622; Fermentas Inc., Ontario, CA, United States). MiR-301b-3p expression was detected by qPCR on TaqMan MicroRNA Assay with cDNA as the template. The reaction of qRT-PCR was as follows: 95^°^C for 2 min, then 45 cycles of 95^°^C for 15 s, 60^°^C for 45 s, 72^°^C for 45 s. U6 was taken as the internal reference to normalize results.

qRT-PCR was performed on ADAMTS9-AS1 and TGFBR2 according to the instruction of TaqMan Gene Expression Assays protocol (Applied Biosystems, Foster City, CA, United States). GAPDH was taken as the internal reference. PCR procedures were designed as follows: 95^°^C for 10 min, 35 cycle of 95^°^C for 15 s, 60^°^C for 30 s, 72^°^C for 45 s. All qRT-PCR was set with 3 parallel wells. Primers were designed as shown in Table 1 and synthesized by TAKARA Company (Beijing, China). Relative transcription level of target gene was formulated by relative quantitative method (2^–△△CT^). △△Ct = △Ct (experimental group)−△Ct (control group), △Ct = Ct (target gene)−t (internal reference), relative transcription level of target mRNA = 2^–△△^^Ct^.

**TABLE 1:** qRT-RCR primer sequences.

Primer sequence	Forward (5′→3′)	Reverse (5′→3′)
ADAMTS9-AS1	ACTCGGTCTCTCT GGCTATT	GGGCTGTTCTCTGT CTTCTTAG
TGFBR2	GTAGCTCTGATGAG TGCAATGAC	CAGATATGGCAACT CCCAGTG
miR-301b-3p	CAGGTGCTCTGAC GAGGTTG	TGGTCCCAGATGC TTTGACA
U6	CTCGCTTCGGCAG CACATA	AACGATTCACGAA TTTGCGT
GAPDH	GAAGGTGAAGG TCGGAGTC	GAAGATGGTGAT GGGATTTC

### Western Blot

Total proteins were acquired from cells (1 × 10^6^ per well), and protein concentration was determined by bicinchoninic acid (BCA) kit (Thermo, United States). Next, 30 μg total proteins were loaded on sodium dodecyl sulfate polyacrylamide gel electrophoresis (SDS-PAGE) at constant 80 V for 35 min and then 120 V for 45 min. The proteins were transferred onto a polyvinylidene fluoride (PVDF) membrane (Amersham, United States). The membrane was blocked with 5% skim milk at normal temperature for 1 h. It was followed by an overnight incubation at 4°C overnight with rabbit anti-TGFBR2, Ki67, PCNA, MMP-2, MMP-9, JAK1, p-JAK1, JAK2, p-JAK2, STAT1, p-STAT1, STAT3, p-STAT3, β-actin. All antibodies were offered by Abcam (Cambridge, United Kingdom). The membrane was washed with Tris-buffered saline with 0.1% Tween^®^20 detergent (TBST) three times (10 min each time). Afterward, secondary antibody goat anti-rabbit IgG H&L labeled by horseradish peroxidase (HRP) was added for 1 h of incubation at room temperature. Phosphate buffered saline with Tween 20 (PBST) solution was used for 3 washes of the membrane (each time 10 min). Chemiluminescence apparatus (GE, United States) was applied for scanning and development. Protein bands were gray-scale scanned with Image Pro Plus 6.0 (Media Cybernetics, United States) software to analyze relative protein expression.

### *In vitro* Colony Formation Assay

The transfected breast cancer cells (1 × 10^4^ per well) were inoculated into 12-well plates and cultured at 37^°^C with 5% CO_2_ for 2 weeks. Then cells were fixed with 4% paraformaldehyde and stained with 0.1% crystal violet. The number of colonies, whose cells are more than 50, was manually counted.

### EdU Assay

Cells (3 × 10^5^ per well) to be tested were seeded in 24-well plates with 3 parallel wells. EdU (C10310-1, RiboBio, Guangzhou, China) was added into the culture solution to achieve a concentration of 10 μmol/l for 2 h of incubation in the incubator. After removing medium, cells undergone 15 min of fixation with PBS + 4% paraformaldehyde at common temperature for and twice washes with PBS + 3% bovine serum albumin (BSA). PBS containing 0.5% Triton-100 was added for 20 min of incubation at room temperature and then PBS with 3% BSA was used to wash cells twice. Each well was added with 100 μl Apollo^®^ 567 (C10310-1, RiboBio, Guangzhou, China) for 30 min of incubation in the dark at room temperature. Thereafter, cells were washed with PBS containing 3% BSA. DIPA (C0065, Solarbio, Beijing, China) was added to stain nucleus for 5 min. Next, cells were washed with PBS in triplicate and sealed. Cells were observed under a fluorescent microscope (Type: FM-600, Shanghai PuDa Company) and positive cell number in each field was recorded. Under the microscope, total cells presented as blue and positive cells presented as red. Percentage of EdU stained positive cells was calculated in 3 random fields of each well. EdU positive rate = (EdU positive nucleus number/total nucleus) × 100%.

### Transwell Assay

Extracellular matrix (ECM) gel was maintained at 4°C overnight and then attenuated with serum-free medium at 1:9 ratio to 1 mg/ml. Each polycarbonate membrane of the 24-well Transwell chamber was added with 40 μl ECM gel and maintained generally in the incubator for 5 h. Polymerized gel was prepared by ECM gel and redundant solution was removed. Then, 70 μl/chamber pure Dulbecco’s Modified Eagle Medium (DMEM) was added. Cells (1 × 10^4^ per well) were incubated in an incubator at 37^°^C for 0.5 h to rehydrate Matrigel and redundant culture solution was removed. Cells in each group were starved for 24 h without serum, digested, centrifuged and resuspended with DMEM without FBS to a final concentration of 2.5 × 10^5^ cells/ml. The upper chamber where the basement membrane was hydrated was added with added 0.2 ml suspension, while the lower chamber was added with 700 μl pre-cooled DMEM containing 10% FBS. Then, plates were generally cultured in a moist incubator for 24 h. Thereafter, the chamber was discarded, and cells in the chamber and on the membrane were wiped by a wet cotton swab. The remaining cells were fixed with methyl alcohol for 30 min, stained with 0.1% crystal violet for 20 min and dried in inverted position. Cells were observed and photographed under an inverted microscope. Average number of cells that passed through the membrane was counted in 5 random fields.

### RNA Binding Protein Immunoprecipitation

Co-transfection was, respectively, undertaken on MCD-7 cells (3 × 10^5^ per well) with mimic NC/miR-301b-3p mimic along with oe-NC/oe-ADAMTS9-AS1. The binding between miR-301b-3p with ADAMTS9-AS1 and TGFBR2 was examined using RIP kit. MCF-7 cells were washed with pre-cooled PBS and then the supernatant was removed. Cells were lysed with equivalent volume of lysis solution (P0013B, Beyotime) in ice-bath for 5 min and centrifuged at 4^°^C and 14,000 rpm for 10 min. The cell extract was incubated with antibodies for co-precipitation. Specific steps were: 50 μl magnetic beads were taken from each co-precipitation reaction system, washed and resuspended in 100 μl RIP Wash Buffer. According to experimental groups, 5 μg antibodies were added for binding. Magnetic beads-antibody complex was resuspended in 900 μl RIP Wash Buffer after being washed. Thereafter, 100 μl cell extract was added for an overnight incubation at 4^°^C. Samples were placed on the magnetic drill to collect magnetic beads-protein complex. The antibodies used in RIP were: Ago2 (ab32381, 1:50, Abcam, United Kingdom) which was evenly shaken at room temperature for 30 min, and IgG (1:100, ab172730, Abcam, United Kingdom) as the NC. Samples were digested by protease K. Then, RNA was extracted from the samples for succeeding analyses.

### Dual-Luciferase Reporter Gene Assay

The whole length of 3′UTR of TGFBR2 gene was amplified. PCR products were cloned into multiple cloning sites on the downstream of pmirGLO luciferase gene (Promega, WI, United States) by using restriction enzyme sites, to construct TGFBR2-wild type (WT) (CCAGCUAUGA CCACAUUGCACUU) group. Binding sites of miR-301b-3p and its target gene were predicted by target gene databases. TGFBR2-mutant (MUT) vector (CCAGCUAUGACCACAUAC GUGAU) was constructed by PCR site-directed mutation method. Renilla luciferase expression vector pRL-TK (TaKaRa, Dalian, China) was taken as the internal reference. HEK-293T cells (3 × 10^5^ per well) were co-transfected with mimic NC/miR-301b-3p mimic and luciferase reporter vectors, respectively. Dual Luciferase activity was tested by using Dual-Luciferase Reporter Assay System (Promega, Madison, WI, United States).

### Statistical Analysis

Results were treated with SPSS 21.0 statistical software (SPSS, Inc., Chicago, IL, United States). Three times of biological repetitions and technical repetitions were required for each assay. Measurement data were shown as mean ± standard deviation. Comparison of two or more groups were, respectively, Student’s *t*-test and analysis of variance (ANOVA). Repeated-measures ANOVA was adopted to compare results at each time point. Patient’s overall survival curve was calculated by Kaplan-Meier. Patient’s survival differences were analyzed by log-rank. Analysis method of enumeration data was chi-square test. *P <* 0.05 stands for statistical significance.

## Results

### Inactivated ADAMTS9-AS1 in Breast Cancer

**FIGURE 1:**
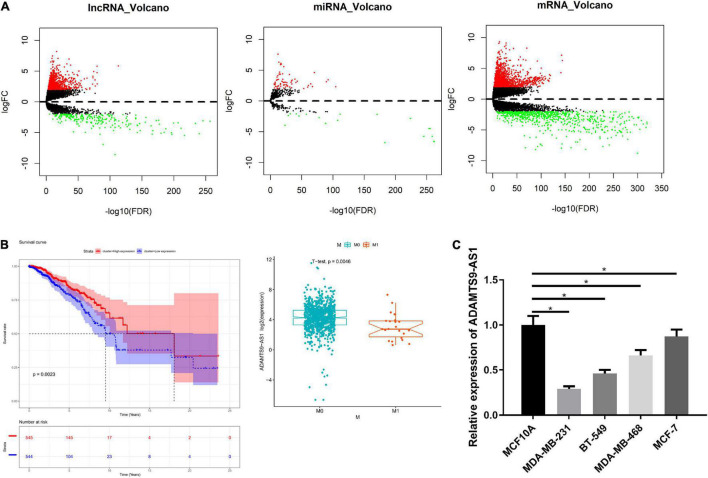
ADAMTS9-AS1 is lowly expressed in breast cancer. **(A)** Volcano map of DElncRNAs, DEmiRNAs and DEmRNAs in breast cancer tumor group and normal group. **(B)** The survival curve of ADAMTS9-AS1 expression level on the prognosis of patients, with *X*-axis representing time (in years) and *Y*-axis representing survival. Red: high expression, blue: low expression. **(C)** The level of ADAMTS9-AS1 in breast cancer cells; *p < 0.05.

There were 1,017 DElncRNAs, 74 DEmiRNAs and 2,161 DEmRNAs, as offered by Figure 1A and detailed in Supplementary Tables 2–4. miRcode database was used to compare the results, finding that 60 lncRNAs and 18 miRNAs had binding sites (Supplementary Table 5). Low and high ADAMTS9-AS1 groups were classified by median ADAMTS9-AS1 level. Survival analysis of the 60 lncRNAs revealed notably lower survival of low ADAMTS9-AS1 expression group. Furthermore, ADAMTS9-AS1 levels noticeably pertained to clinical M stage, and patients in high ADAMTS9-AS1 group were mostly in M0 stage (Figure 1B). Hence the downregulation of ADAMTS9-AS1 remarkably impacted patient’s prognostic conditions. Meanwhile, qRT-PCR uncovered that level of ADAMTS9-AS1 was prominently low in cancer cell lines. MDA-MB-231 cells (*p* < 0.05) and MCF-7 cells (*p* < 0.05) showed the lowest and highest ADAMTS9-AS1 levels, respectively (Figure 1C).

### ADAMTS9-AS1 Restrains Breast Cancer Cell Proliferation and Invasion

**FIGURE 2:**
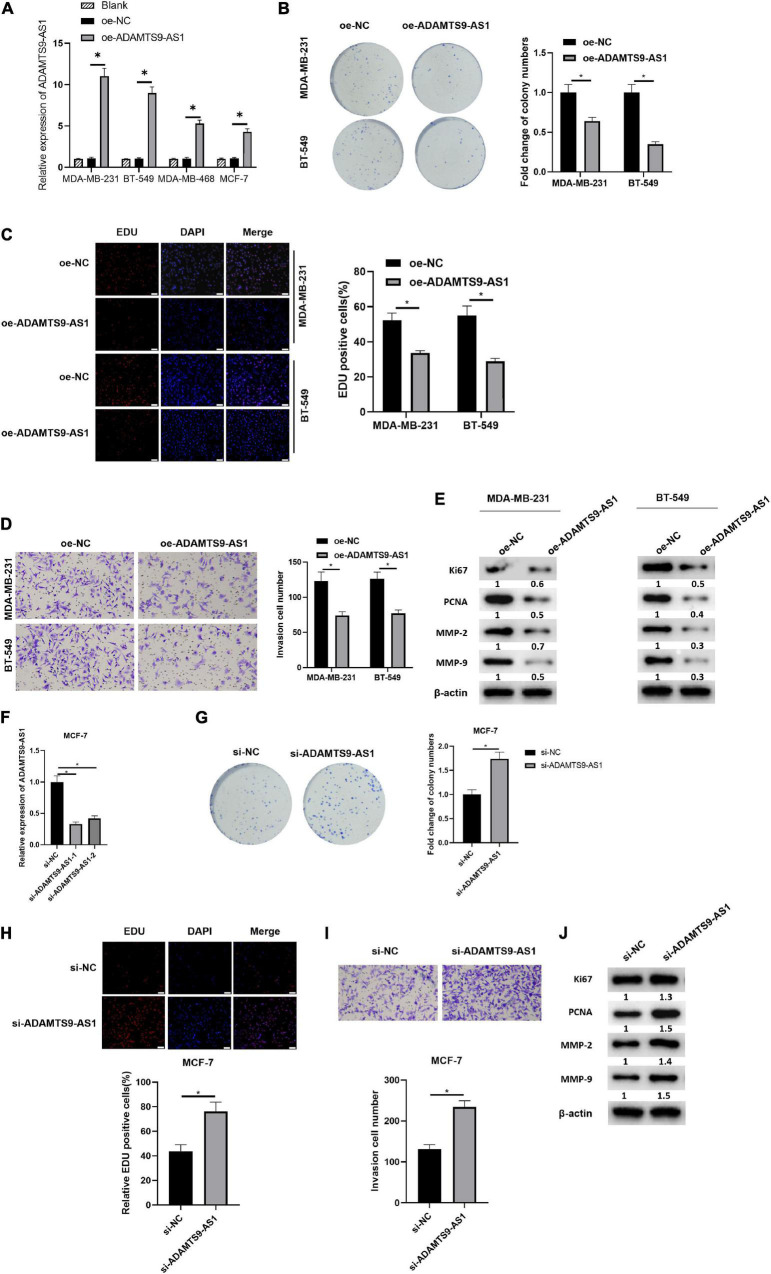
ADAMTS9-AS1 restrains cancer cell invasion and proliferation. ADAMTS9-AS1 was overexpressed in MDA-MB-231 and BT-549, while being silenced in MCF7. **(A)** Level of ADAMTS9-AS1 in breast cancer cells. **(B)** Results of *in vitro* colony formation method. **(C)** Results of EdU method (400×). **(D)** Cell invasion results (100×). **(E)** The expression of Ki67, PCNA, MMP-2 and MMP-9 after interfering with ADAMTS9-AS1 expression. **(F)** ADAMTS9-AS1 level in cancer cells. **(G)** Proliferation of cancer cells. **(H)** Results of EdU method (400×). **(I)** Transwell method is used to test cell invasion (100×). **(J)** The expression of Ki67, PCNA, MMP-2 and MMP-9. *p < 0.05.

After transfection of plasmids oe-NC and oe-ADAMTS9-AS1 was done, the latter exhibited a markedly increase in ADAMTS9-AS1 level, with respect to the former group (Figure 2A). Transfected cells were therefore employed in the succeeding analyses. Cell proliferation was examined through colony formation assay and EdU assay (Figures 2B,C). The results revealed that the cell proliferation was remarkably reduced by overexpressed ADAMTS9-AS1. Meanwhile, the result of Transwell assay uncovered that the overexpressed ADAMTS9-AS1 notably constrained the invasive ability of cells (Figure 2D). Western blot result revealed that oe-ADAMTS9-AS1 markedly decreased the expression of tumor markers for proliferation and invasion, such as Ki67, PCNA, MMP-2, MMP-9 (Figure 2E), suggesting that oe-ADAMTS9-AS1 could reduce tumor proliferative, and invasive abilities.

ADAMTS9-AS1 was silenced in MCF-7 and the expression was examined afterward, finding a prominent reduction in si-ADAMTS9-AS1-1/si-ADAMTS9-AS1-2 groups (Figure 2F). The si-ADAMTS9-AS1-1 with the highest interference efficiency was employed hereinafter and was uniformly named as si-ADAMTS9-AS1. Further, the changes of breast cancer proliferative ability (Figures 2G,H) and invasive ability (Figure 2I) were examined by multiple methods. The results uncovered that cancer cell proliferation and invasion were prominently boosted after the downregulation of ADAMTS9-AS1. Western blot result uncovered that levels of Ki67, MMP-9, MMP-2 and PCNA were notably boosted after the downregulation of ADAMTS9-AS1 (Figure 2J). All the above, ADAMTS9-AS1 repressed the proliferation and invasion of breast cancer cells. Morphology of cells after ADAMTS9-AS1 silence or overexpression manifested no noticeable differences between groups (Supplementary Figure 2).

### ADAMTS9-AS1 Traps miR-301b-3p to Regulate TGFBR2

**FIGURE 3:**
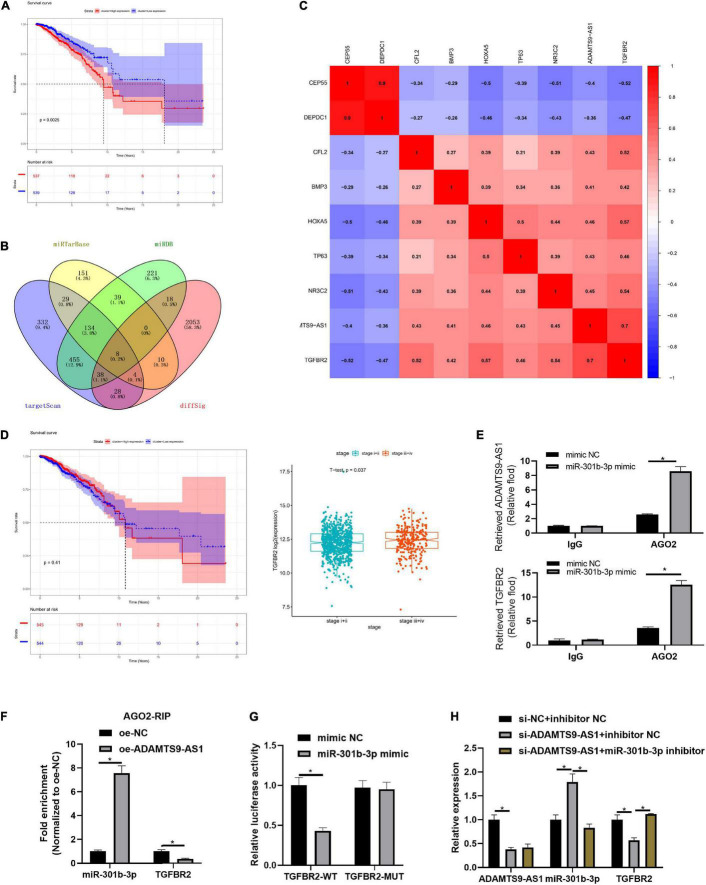
ADAMTS9-AS1 competitively binds to miR-301b-3p to promote TGFBR2. **(A)** Survival curve of miR-301b-3p level and prognosis of patients. **(B)** Venn plot of predicted mRNAs and DEmRNAs; diffSig denotes differentially expressed genes screened from TCGA database. **(C)** Association between ADAMTS9-AS1 and 8 target mRNAs. **(D)** The survival curve of TGFBR2 expression level on the prognosis of patients. **(E,F)** Results of RIP method. **(G)** Result of dual-luciferase method. **(H)** The expression of ADAMTS9-AS1, miR-301b-3p and TGFBR2. *p < 0.05.

ADAMTS9-AS1 targeted 6 DEmiRNAs (miR-301b-3p, miR-96-3p, miR-144-3p, miR-145-3p, miR-182-3p, and miR-21-3p), among which miR-301b-3p markedly pertained to patient’s prognosis (Figure 3A). Median miR-301b-3p was utilized to classify groups. As displayed in Supplementary Figure 1, miR-301b-3p levels were irrelevant to TNM stage. 8 DEmRNAs binding with miR-301b-3p were discovered in the overlap of target genes and DEmRNAs (Figure 3B). These 8 genes were subjected to Pearson correlation. Pearson correlation coefficient between TGFBR2 and ADAMTS9-AS1 was the highest (Figure 3C). Median TGFBR2 level was utilized for grouping, followed by survival analysis and expression differences. Level of TGFBR2 was of notably difference in varying pathological stages (Figure 3D). Moreover, TGFBR2 participates in the proliferation and invasion of tumor cells (Liu et al., 2018; Zhou B. et al., 2018). Therefore, we speculated that ADAMTS9-AS1 mediated TGFBR2 levels via sequestering miR-301b-3p, thus regulating cancer proliferation and invasion.

Further mechanism verification was conducted. RIP assay exhibited stimulated enrichment of ADAMTS9-AS1/TGFBR2 upon miR-301b-3p mimic treatment, implying that miR-301b-3p bound to ADAMTS9-AS1 and TGFBR2, respectively (*p* < 0.05) (Figure 3E). Moreover, similar trend was observed concerning enrichment of miR-301b-3p/TGFBR2 in MCF-7 cell with oe-ADAMTS9-AS1, implying that ADAMTS9-AS1/TGFBR2 competitively bound miR-301b-3p (Figure 3F). Dual-luciferase result uncovered that miR-301b-3p mimic markedly repressed luciferase intensity of TGFBR2-WT (*p* < 0.05) but did not impact TGFBR2-MUT (Figure 3G). TGFBR2 expression was prominently decreased after ADAMTS9-AS1 was interfered (*p* < 0.05), while miR-301b-3p inhibitor could reverse TGFBR2 expression (*p* < 0.05) (Figure 3H). Altogether, ADAMTS9-AS1 competitively bound miR-301b-3p, so as to promote the expression of TGFBR2.

### ADAMTS9-AS1 Traps miR-301b-3p to Regulate TGFBR2, Thereby Repressing Breast Cancer Cell Proliferation and Invasion

**FIGURE 4:**
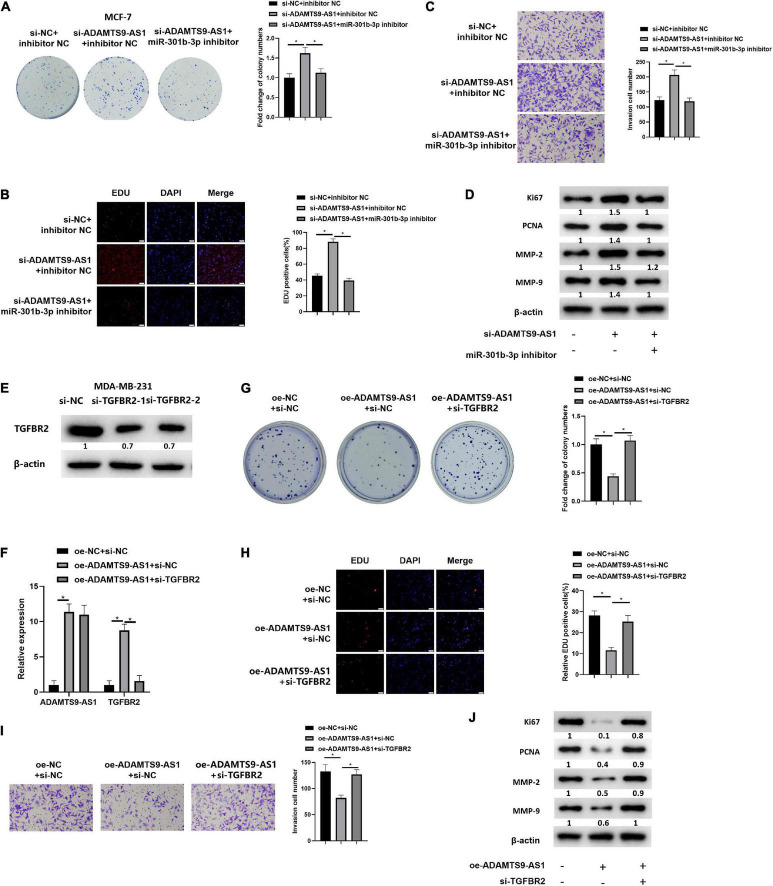
ADAMTS9-AS1 promotes TGFBR2 through miR-301b-3p to inhibit breast cancer cell invasion and proliferation. **(A)** The proliferation of MCF-7 cells. **(B)** EdU assay used to detect MCF-7 cell proliferation (400×). **(C)** Invasion of MCF-7 cells (100×). **(D)** Levels of Ki67, PCNA, MMP-2 and MMP-9. **(E)** Level of TGFBR2 in si-NC, si-TGFBR2-1 and si-TGFBR2-2 groups. The silencing sequence with the highest silencing efficiency was named as si-TGFBR2. **(F)** ADAMTS9-AS1 and TGFBR2 expression in MDA-MB-231 cells. **(G)** Proliferation of MDA-MB-231. **(H)** Proliferation of MDA-MB-231 (400×). **(I)** Cancer cell invasion (100×). **(J)** Levels of Ki67, PCNA, MMP-2 and MMP-9. *p < 0.05.

We first divided MCF-7 cells into varying transfecting groups. Further, changes in cell proliferation (Figures 4A,B) and invasion (Figure 4C) of breast cancer cells were detected by *in vitro* colony formation, EdU and Transwell assays, respectively. The results revealed that the invasion (*p* < 0.05) as well as proliferation (*p* < 0.05) of breast cancer were markedly boosted after the downregulation of ADAMTS9-AS1 expression, while these abilities reversed upon co-transfection with si-ADAMTS9-AS1 and miR-301b-3p inhibitor. Western blot result uncovered that Ki67, MMP-9, MMP-2, PCNA and other tumor markers were prominently activated after the downregulation of ADAMTS9-AS1, indicating increased tumor proliferative activity. However, in si-ADAMTS9-AS1 + miR-301b-3p inhibitor group, the expression of those tumor makers was reversed (*p* < 0.05) (Figure 4D), indicating a reduced tumor proliferative activity. To sum up, downregulated ADAMTS9-AS1 facilitated cancer cell invasion and proliferation through miR-301b-3p.

Two TGFBR2 interference sequences were constructed, and TGFBR2 expression was examined. The silencing sequence with the highest silencing efficiency, si-TGFBR2-2 (*p* < 0.05), was selected and named as si-TGFBR2 (Figure 4E). Three transfecting groups were generated. qRT-PCR indicated that TGFBR2 level was notably boosted after the overexpression of ADAMTS9-AS1 (*p* < 0.05). Relative to the oe-ADAMTS9-AS1 + si-NC group, the level of ADAMTS9-AS1 in the oe-ADAMTS9-AS1 + si-TGFBR2 group had no notable change (*p* < 0.05) while TGFBR2 was prominently constrained (*p* < 0.05) (Figure 4F). Further, cell proliferation (Figures 4G,H) and invasion (Figure 4I) were detected to be markedly reduced upon ADAMTS9-AS1 overexpression(*p* < 0.05), while overexpressed ADAMTS9-AS1 and silencing TGFBR2 together could reverse that effect (*p* < 0.05). Western blot result uncovered that Ki67, PCNA, MMP-2, and MMP-9 were notably repressed upon ADAMTS9-AS1 overexpression (*p* < 0.05), indicating that the tumor proliferation was decreased. However, overexpressed ADAMTS9-AS1 and silencing TGFBR2 together could reverse this regulatory effect (*p* < 0.05), indicating increased tumor proliferation (Figure 4J). Altogether, ADAMTS9-AS1 restrained the proliferation and invasion of breast cancer via stimulating the level of TGFBR2 through miR-301b-3p.

### ADAMTS9-AS1 Traps miR-301b-3p to Target TGFBR2, Thereby Regulating the JAK STAT Signaling Pathway

**FIGURE 5:**
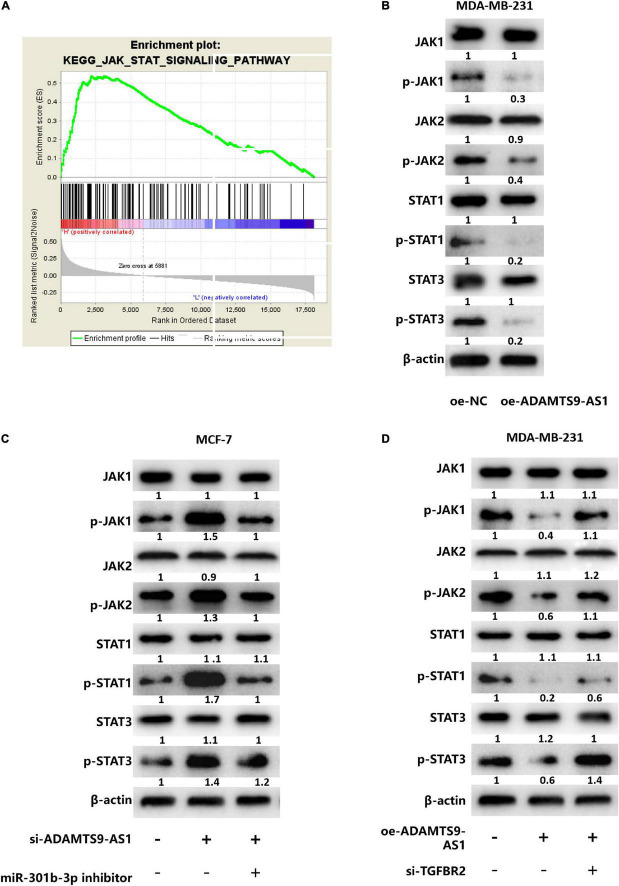
ADAMTS9-AS1 regulates the JAK STAT signaling pathway by targeting TGFBR2 through miR-301b-3p. **(A)** GSEA enrichment pathway of TGFBR2 (JAK STAT signaling pathway). **(B)** The expression of JAK STAT signaling-relevant proteins. **(C)** The expression of JAK STAT signaling-relevant proteins in si-NC + inhibitor NC, si-ADAMTS9-AS1 + inhibitor NC and si-ADAMTS9-AS1 + miR-301b-3p inhibitor. **(D)** Levels of JAK STAT signaling-relevant proteins in oe-NC + si-NC, oe-ADAMTS9-AS1 + si-NC and oe-ADAMTS9-AS1 + si-TGFBR2.

GSEA analysis result revealed that TGFBR2 was notably enriched in the JAK STAT signaling (Figure 5A), suggesting that ADAMTS9-AS1/miR-301b-3p/TGFBR2 signaling axis may regulate breast cancer cells through the JAK STAT signaling pathway. For further verification, ADAMTS9-AS1 was firstly overexpressed in cell line MDA-MB-231. Corresponding detection uncovered that the protein levels of p-STAT3, p-JAK2, p-STAT1 and p-JAK1 in the oe-ADAMTS9-AS1 group were notably repressed (*p* < 0.05) (Figure 5B), implying that ADAMTS9-AS1 constrained the JAK STAT signaling pathway. Further analyses depicted that the protein levels of p-STAT3, p-STAT1, p-JAK2, and p-JAK1 were notably boosted after silencing ADAMTS9-AS1, while the regulatory effects were reversed in si-ADAMTS9-AS1 + miR-301b-3p inhibitor (*p* < 0.05) (Figure 5C). Generally, silencing ADAMTS9-AS1 activated the JAK STAT signaling pathway, while silencing miR-301b-3p could reverse this impact.

MDA-MB-231 cells divided into the following transfecting groups: oe-NC + si-NC, oe-ADAMTS9-AS1 + si-NC and oe-ADAMTS9-AS1 + si-TGFBR2. Western blot examined that p-JAK2, p-STAT3, p-JAK1 and p-STAT1 protein levels were notably reduced in oe-ADAMTS9-AS1 + si-NC group, while co-transfection of oe-ADAMTS9-AS1 and si-TGFBR2 reversed the regulatory effect caused by overexpressed ADAMTS9-AS1 (*p* < 0.05) (Figure 5D), suggesting that ADAMTS9-AS1 inhibited the JAK STAT signaling pathway, while silencing TGFBR2 reversed that inhibitory effect. Overall, ADAMTS9-AS1 regulated the JAK STAT signaling pathway by targeting TGFBR2 through miR-301b-3p.

### ADAMTS9-AS1 Constrains Breast Cancer Cell Proliferation and Invasion via Regulating JAK STAT Signaling Pathway

**FIGURE 6:**
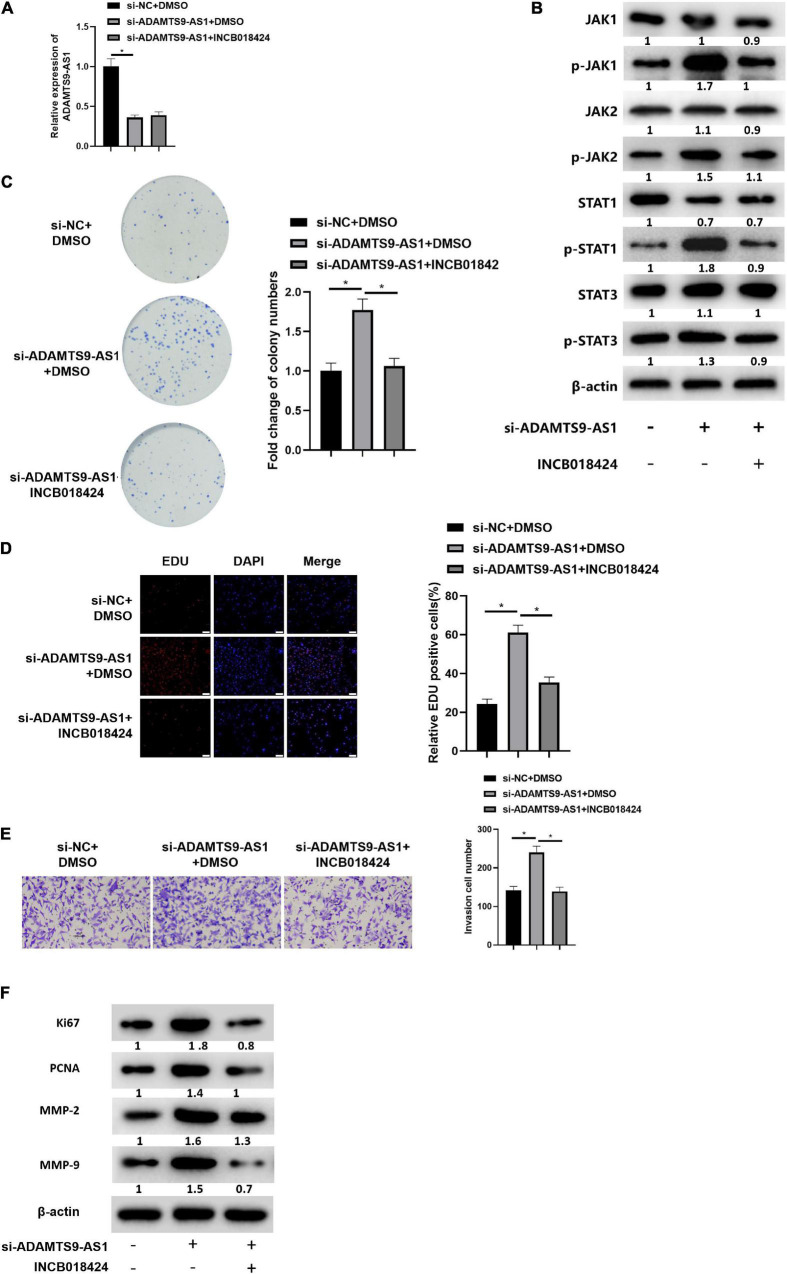
ADAMTS9-AS1 represses cancer invasion and proliferation by regulating JAK STAT signaling pathway. **(A)** ADAMTS9-AS1 expression. **(B)** Levels of JAK STAT signaling-relevant proteins. **(C)** The proliferation of breast cancer cells *in vitro*. **(D)** The proliferation of breast cancer cells (400×). **(E)** Cancer cell invasion (100×). **(F)** Levels of Ki67, PCNA, MMP-2 and MMP-9. *p < 0.05.

MCF-7 cells were simultaneously transfected with si-NC and si-ADAMTS9-AS1, and treated with JAK STAT signaling pathway inhibitor INCB018424 (HY-50856, MedchemExpress; Punwani et al., 2012). qRT-PCR result indicated that the expression of ADAMTS9-AS1 in si-ADAMTS9-AS1 + DMSO group was markedly lower than that in si-NC + DMSO group (*p* < 0.05). While there was no significant difference in ADAMTS9-AS1 expression between the si-ADAMTS9-AS1 + DMSO group and the si-ADAMTS9-AS1 + INCB018424 group (Figure 6A). The expression of JAK STAT signaling pathway-related proteins was detected by western blot. The result suggested that compared with si-NC + DMSO group, the protein expression levels of p-JAK1, p-JAK2, p-STAT1 and p-STAT3 in si-ADAMTS9-AS1 + DMSO group were remarkably increased. Compared with si-ADAMTS9-AS1 + DMSO group, the protein expression levels in si-ADAMTS9-AS1 + INCB018424 group were notably decreased (*p* < 0.05) (Figure 6B). *In vitro* colony formation (Figure 6C), EdU (Figure 6D), and Transwell (Figure 6E) assays indicated that compared with the si-NC + DMSO group, the proliferative ability and invasive ability of breast cancer cells in the si-ADAMTS9-AS1 + DMSO group were prominently increased. Compared with the si-ADAMTS9-AS1 + DMSO group, the proliferative and invasive abilities of breast cancer cells in si-ADAMTS9-AS1 + INCB018424 group were markedly decreased (*p* < 0.05). Western blot result exhibited that compared with si-NC + DMSO group, the expression levels of Ki67, PCNA, MMP-2 and MMP-9 in si-ADAMTS9-AS1 + DMSO group were markedly boosted, indicating increased tumor proliferative activity. Compared with si-ADAMTS9-AS1 + DMSO group, the expression levels of Ki67, PCNA, MMP-2 and MMP-9 in si-ADAMTS9-AS1 + INCB018424 group were prominently constrained, indicating decreased tumor proliferative activity (*p* < 0.05) (Figure 6F). In general, ADAMTS9-AS1 could suppress the proliferation and invasion of breast cancer cells by regulating the JAK STAT signaling pathway. Besides, JAK inhibitor did not impact TGFBR2 levels according to detection results (Supplementary Figure 3).

## Discussion

Exposed to metastasis and recurrence, patients with breast cancer bear unsatisfied therapy efficacy and survival. Mechanism for invasion and proliferation is therefore urgently warranted for depiction. ADAMTS9-AS1 was noticeably inactivated in breast cancer cells here. ADAMTS9-AS1 is a dysregulated antisense lncRNA in many tumors and is involved in the pathogenesis of a variety of human malignancies (Wang et al., 2016; Xing et al., 2018). For example, in colorectal cancer, lncRNA ADAMTS9-AS1 constrains invasion and migration of colorectal cancer (Li et al., 2020). ADAMTS9-AS1 also represses tumor-relevant prostate cancer behaviors (Zhou et al., 2021). The present paper found that ADAMTS9-AS1 could repress cancer proliferation and invasion through *in vitro* functional experiments. Nevertheless, there are reports that are contrary to our research results. For instance, overexpression of ADAMTS9-AS1 aggravates hepatocellular carcinoma (Zhang et al., 2020) and colorectal cancer development (Chen et al., 2020). We speculated that the different roles of ADAMTS9-A1 may be attributed to different cancer types.

To date, lncRNA/miRNA axis has been a limelight in the field of cancer. LncRNA can trap miRNA, regulate mRNA, and modulate progression of various cancers (Ao et al., 2019; Tu et al., 2019). This paper verified miR-301b-3p/ADAMTS9-AS1/TGFBR2 interaction by bioinformatics method, RIP and dual luciferase assay. This paper discovered relevance of miR-301b-3p and poor prognosis of breast cancer patients, and the cancer-promoting effect of si-ADAMTS9-AS1 could be reversed when cells with miR-301b-3p inhibitor at the same time. TGFBR2 was reported as a functional mRNA. For instance, TGFBR2 down-regulation will promote the malignant progression of prostate cancer (Zhou H. et al., 2018). MiR-3139 targets TGFBR2 expression to promote cell migration and invasion in colorectal cancer (He et al., 2019). Here, inhibiting the expression of TGFBR2 could weaken the tumor suppressor effect of ADAMTS9-AS1, suggesting that TGFBR2 was an inhibitor in breast cancer, which is similar to the results of previous studies.

Subsequently, we conducted enrichment analysis on TGFBR2 and found that it was mainly enriched in the JAK STAT signaling. The STAT3 protein is widely present in a variety of tumor tissue, affecting many biological behaviors of tumor cells, and acting as an oncogene, while JAK is responsible for the activation of STAT (Bournazou and Bromberg, 2013; Panni et al., 2014; Wen et al., 2015). Dysregulation of JAK STAT signaling pertains to breast cancer metastasis along with high risk of recurrence (Khanna et al., 2018). In addition, activated STAT3 triggers characteristics of cancer, including angiogenesis (Chung et al., 2014; Banerjee and Resat, 2016). Herein, in combination with rescue experiment, we found that ADAMTS9-AS1 constrained the activation of the JAK STAT signaling through miR-301b-3p/TGFBR2 axis. Thus, cancer invasion and proliferation were repressed.

The paper confirmed that ADAMTS9-AS1 was inactivated in breast cancer cells. ADAMTS9-AS1 could sequester miR-301b-3p to mediate TGFBR2 protein, thereby affecting the JAK STAT signaling, and repressing tumor progression. ADAMTS9-AS1/miR-301b-3p/TGFBR2 regulatory network may provide a more effective clinical treatment strategy for breast cancer patients. ADAMTS9-AS1 might serve as a promising factor for breast cancer. However, this study also has some limitations. First, we lack tissue samples and *in vivo* experiments to fully confirm the accuracy of the results. Second, detailed investigations on the genes that make up the lncRNA ADAMTS9-AS1/miR-301b-3p/TGFBR2 axis can further understand the mechanism of lncRNA ADAMTS9-AS1 inhibiting breast cancer progression. In the future, we will add animal experiments, and also pay more attention to the relationship between ADAMTS9-AS1 and other potential target miRNAs. We hope to offer a more perfect basis for therapeutic strategy of breast cancer.

## Data Availability Statement

The original contributions presented in the study are included in the article/Supplementary Material, further inquiries can be directed to the corresponding author/s.

## Author Contributions

JC contributed to data analysis and manuscript writing. LC contributed to experiment concrete implementation. WZ acquired the data. RW looked up the literature. XW revised the manuscript. ZC designed the study and wrote the manuscript. All authors read and approved the final manuscript.

## Conflict of Interest

The authors declare that the research was conducted in the absence of any commercial or financial relationships that could be construed as a potential conflict of interest.

## Publisher’s Note

All claims expressed in this article are solely those of the authors and do not necessarily represent those of their affiliated organizations, or those of the publisher, the editors and the reviewers. Any product that may be evaluated in this article, or claim that may be made by its manufacturer, is not guaranteed or endorsed by the publisher.

## Footnotes

1
https://portal.gdc.cancer.gov/

2
http://www.mircode.org/

3
http://mirdb.org/

4
http://www.targetscan.org/

5
http://mirtarbase.mbc.nctu.EdU.tw/php/search.php

## Supplementary Material

Supplementary Figure 1 Association between miR-301b-3p and patient’s clinicalstage and TNM stage. **(A)** Box plot of miR-301b-3p expression at different clinical stages; **(B)** Box plot of miR-301b-3p expression at different T stages; **(C)** Box plot of miR-301b-3p expression at different N stages; **(D)** Box plot of miR-301b-3p expression at different M stages.

Supplementary Figure 2 Cell morphology of MCF-7 upon overexpression or silence of ADAMTS9-AS1.

Supplementary Figure 3 TGFBR2 protein and mRNA in MCF-10A and MCF-7 cells.

Supplementary Table 1 Clinical information of patients.

Supplementary Table 2 Detailed information of 1,017 DElncRNAs.

Supplementary Table 3 Detailed information of 74 DEmiRNAs.

Supplementary Table 4 Detailed information of 2,161 DEmRNAs.

Supplementary Table 5 Binding between 60 lncRNA and 18 miRNAs.
