# Supplementary figures and images for: ADAMTS9-AS1 Constrains Breast Cancer Cell Invasion and Proliferation via Sequestering miR-301b-3p
Source: Front Cell Dev Biol. 2021 Nov 24;9:719993. doi: 10.3389/fcell.2021.719993 (PMC8652087; doi:10.3389/fcell.2021.719993)

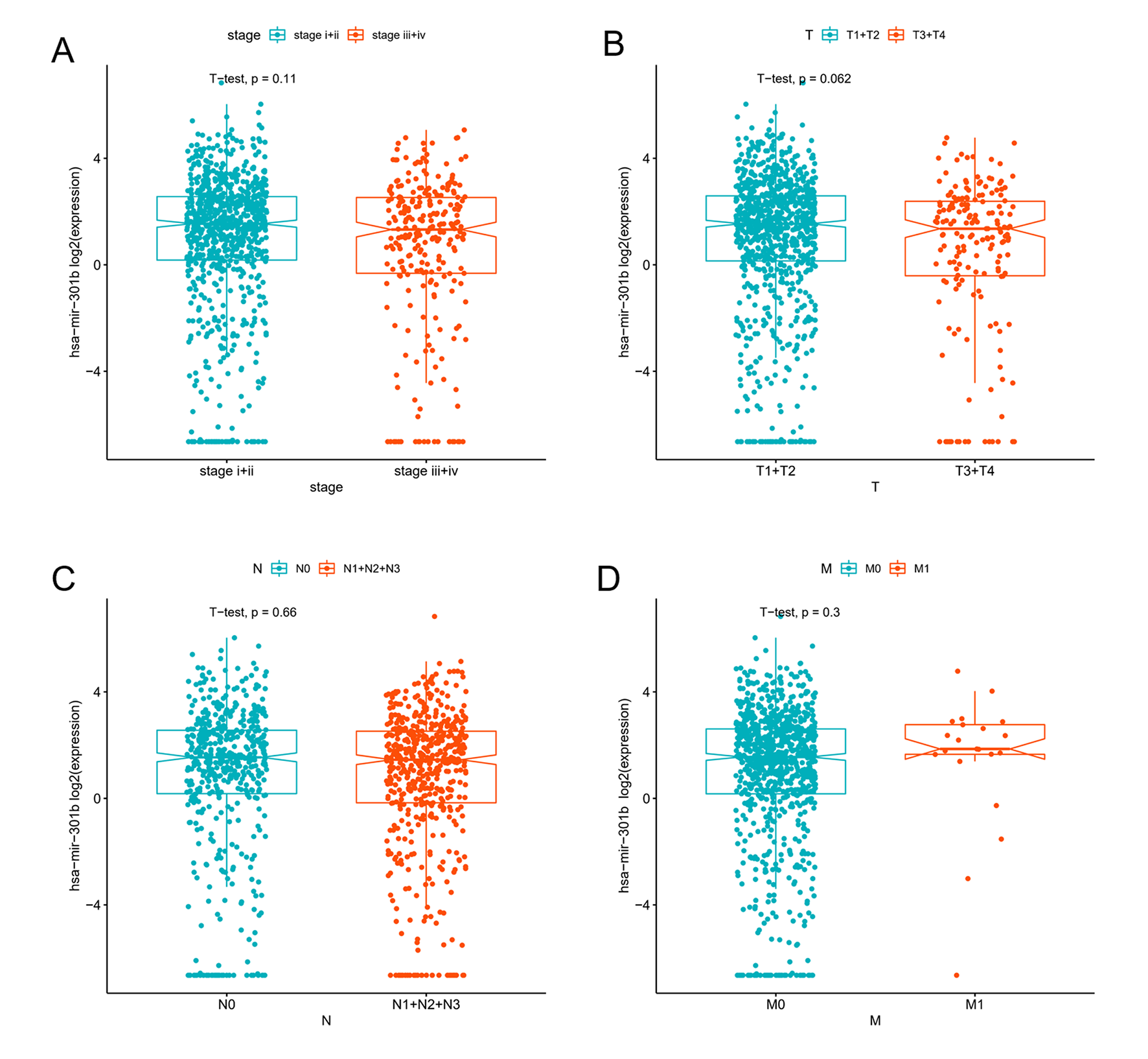

Supplement: Supplementary Figure 1 — Association between miR-301b-3p and patient’s clinicalstage and TNM stage. (A) Box plot of miR-301b-3p expression at different clinical stages; (B) Box plot of miR-301b-3p expression at different T stages; (C) Box plot of miR-301b-3p expression at different N stages; (D) Box plot of miR-301b-3p expression at different M stages. [file Image_1.TIF]

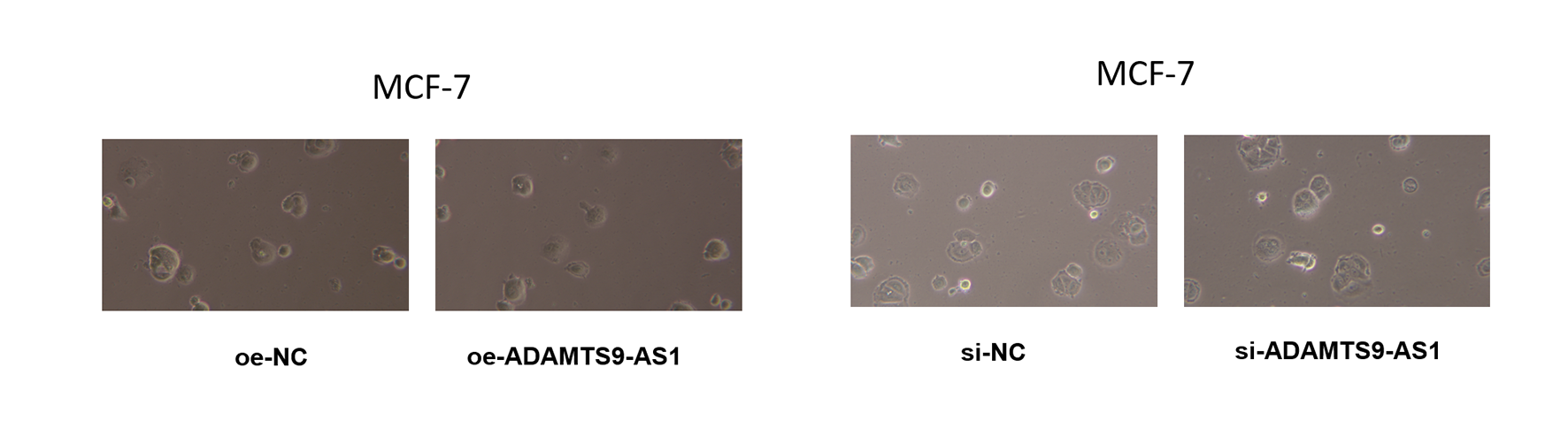

Supplement: Supplementary Figure 2 — Cell morphology of MCF-7 upon overexpression or silence of ADAMTS9-AS1. [file Image_2.TIF]

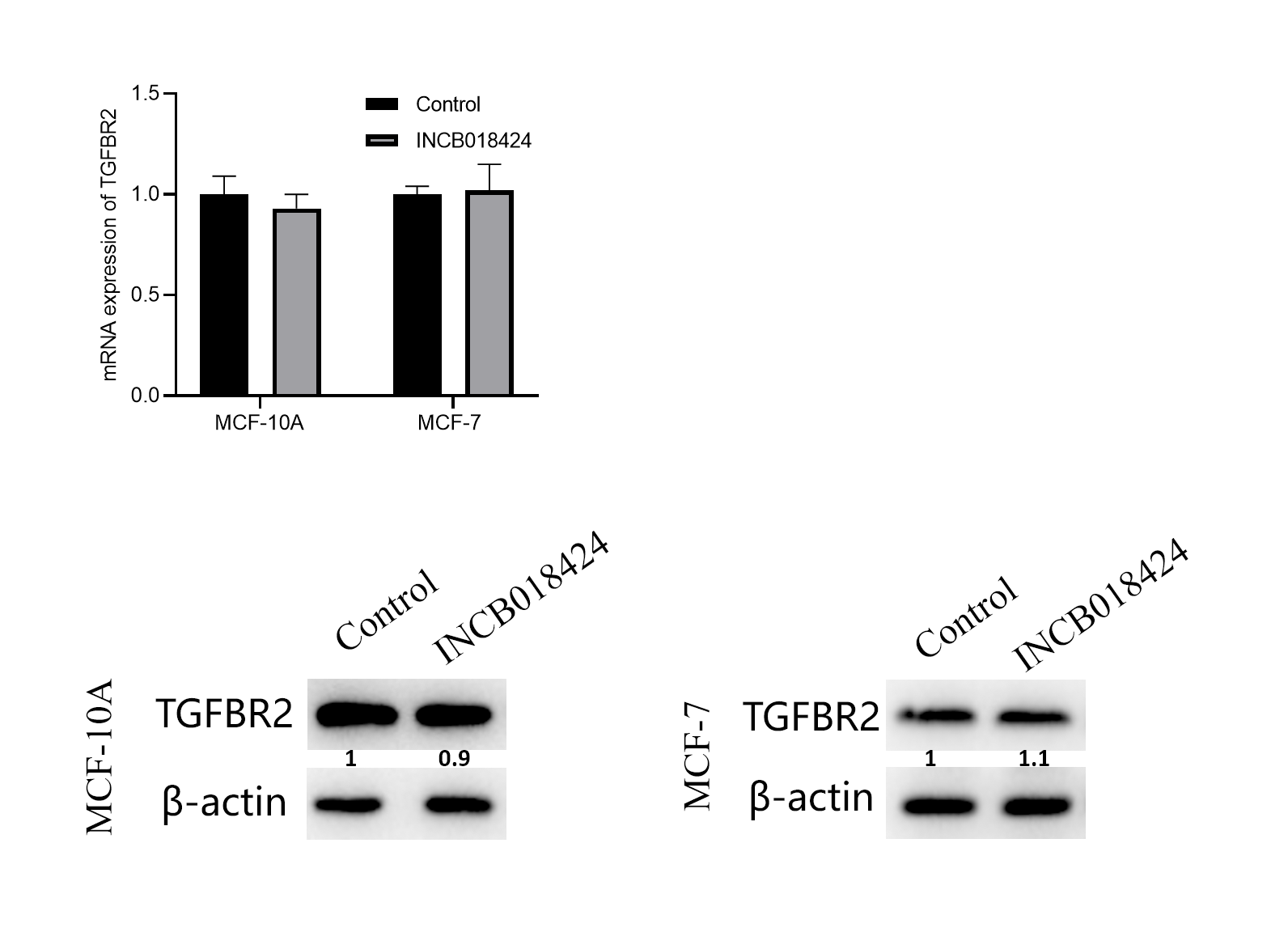

Supplement: Supplementary Figure 3 — TGFBR2 protein and mRNA in MCF-10A and MCF-7 cells. [file Image_3.TIF]
